# Docosahexaenoic Acid Supplemented Diet Influences the Orchidectomy-Induced Vascular Dysfunction in Rat Mesenteric Arteries

**DOI:** 10.1371/journal.pone.0168841

**Published:** 2017-01-09

**Authors:** Diva M. Villalpando, Rocío Navarro, Lara del Campo, Carlota Largo, David Muñoz, María Tabernero, Ramiro Baeza, Cristina Otero, Hugo S. García, Mercedes Ferrer

**Affiliations:** 1 Departamento de Fisiología, Facultad de Medicina, Universidad Autónoma de Madrid, Madrid, Spain; 2 Área Cardiovascular, Instituto de Investigación Hospital Universitario La Paz (IdiPAZ) Madrid, Spain; 3 Cirugía Experimental, Instituto de Investigación Hospital Universitario La Paz (IdiPAZ) Madrid, Spain; 4 Gabinete Veterinario, Facultad de Medicina, Universidad Autónoma de Madrid, Madrid, Spain; 5 Instituto de Catálisis y Petroleoquímica, Consejo Superior de Investigaciones Científicas, Madrid, Spain; 6 Instituto Tecnológico de Veracruz, Veracruz, México; Center for Cancer Research, UNITED STATES

## Abstract

Over the past few decades, the cardiovascular benefits of a high dietary intake of long-chain polyunsaturated fatty acids (PUFAs), like docosahexaenoic acid (DHA), have been extensively studied. However, many of the molecular mechanisms and effects exerted by PUFAs have yet to be well explained. The lack of sex hormones alters vascular tone, and we have described that a DHA-supplemented diet to orchidectomized rats improve vascular function of the aorta. Based on these data and since the mesenteric artery importantly controls the systemic vascular resistance, the objective of this study was to analyze the effect of a DHA-supplemented diet on the mesenteric vascular function from orchidectomized rats. For this purpose mesenteric artery segments obtained from control, orchidectomized or orchidectomized plus DHA-supplemented diet were utilized to analyze: (1) the release of prostanoids, (2) formation of NO and ROS, (3) the vasodilator response to acetylcholine (ACh), as well as the involvement of prostanoids and NO in this response, and (4) the vasoconstrictor response to electrical field stimulation (EFS), analyzing also the effect of exogenous noradrenaline (NA), and the NO donor, sodium nitroprusside (SNP). The results demonstrate beneficial effects of DHA on the vascular function in orchidectomized rats, which include a decrease in the prostanoids release and superoxide formation that were previously augmented by orchidectomy. Additionally, there was an increase in endothelial NO formation and the response to ACh, in which NO involvement and the participation of vasodilator prostanoids were increased. DHA also reversed the decrease in EFS-induced response caused by orchidectomy. All of these findings suggest beneficial effects of DHA on vascular function by reversing the neurogenic response and the endothelial dysfunction caused by orchidectomy.

## Introduction

The involvement of endothelial, hormonal and neural factors in the regulation of vascular function is well established [1, 2], although the contribution of these factors depends on the type of the vessel. In response to different stimuli the endothelium can release different factors, such as nitric oxide (NO), prostanoids, reactive oxygen species (ROS), among others [1]. NO is a signaling molecule formed by the enzyme nitric oxide synthase (NOS) that plays a crucial role in vascular homeostasis regulating the vascular tone, and therefore also influences blood pressure. This molecule exerts vasodilation in smooth muscle cells by stimulating the protein kinase G (PKG) trough soluble guanylate cyclase (sGC) in the smooth muscle of the arterial wall [3]. Also, NO has anti-inflammatory, antithrombotic, antiproliferative, and antioxidant effects. A decrease in NO synthesis and/or bioavailability leads to the development of vascular dysfunction [4].

The endothelium is also a source of ROS generated through the activation of xanthine oxidase, cyclooxygenase, and cytochrome P-450 [5, 6]. Excessive production of ROS, causes vascular dysfunction by outstripping endogenous antioxidant defense mechanisms, and it has been implicated in the pathogenesis of many cardiovascular diseases, including hypercholesterolemia, atherosclerosis, hypertension, diabetes, and heart failure [7].

The vascular tone is also regulated by prostanoids originating from arachidonic acid metabolism through the cyclooxygenase (COX) pathway [8]. Prostanoids are involved in platelet aggregation and inflammation, playing an important role in the regulation of vascular tone in physiopathological conditions. Vasodilator prostanoids such as prostacyclin and prostaglandin E_2_ (PGE_2_) play a role in parallel with NO in the regulation of vascular tone and blood pressure. In comparison, TXA_2_ is considered a potent vasoconstrictor and increased production of this factor is correlated with alterations in vascular functions [9].

Rat mesenteric artery possesses nitrergic and sympathetic innervations in which the release of NO and noradrenaline (NA) are involved in the neuronal regulation of vascular tone [10–12]. Upon release, NO induces vasodilator action as commented above. NA release causes vasoconstrictor effect through the activation of alpha-adrenoceptors [13]. The involvement of sensory innervation in the regulation of vascular tone depends on gender, physiological situation, and even rat strain [13–15].

In addition to the endothelial and neuronal factors, hormones also participate in the control of vascular function. Regarding sex hormones, cardioprotective effects have been reported in men and women [2, 16]. Previous studies from our group have shown that the loss of gonadal function in male and female rats increased the release and function of vasoconstrictor prostanoids [17, 18], as well as the synthesis of ROS [13, 19, 20]. This accumulating production, when maintained for a significant amount of time, could lead to the development of cardiovascular diseases.

On the other hand, several studies have demonstrated the cardiovascular benefits of n-3 polyunsaturated fatty acids (n-3 PUFAs) [21, 22]. The most prominent *n*-3 fatty acids with demonstrated cardiovascular benefits are eicosapentaenoic acid (EPA) and docosahexaenoic acid (DHA), which are predominantly found in fish oils. The mechanisms by which these n-3 PUFAs decrease endothelial dysfunction involve lipid and prostanoid metabolism, leading to secondary favorable effects on blood pressure and thrombosis [23]. Additionally, we have reported the beneficial effect of a DHA-supplemented diet of orchidectomized rats on aorta function [24].

Considering all of these data and taking into account that mesenteric circulation has an important participation in systemic blood pressure control, it would be important to study the influence of PUFAs, specifically DHA, to prevent the functional alterations of mesenteric arteries observed after deprivation of male sex hormones. Therefore, the aim of this work was to study how a DHA-supplemented diet influences the mesenteric artery vascular function from orchidectomized rats, by monitoring: (1) the basal production of NO, ROS and prostanoids, (3) the vasodilator response to acetylcholine (ACh), as well as the involvement of prostanoids and NO in this response (4) the vasoconstrictor response to electrical field stimulation (EFS); additionally, the effect of exogenous NA, and the NO donor, SNP were also analyzed.

## Materials and Methods

### Animals, diets and experimental groups

The protocol was approved by the Animal Ethics Committee of the Universidad Autónoma de Madrid (Ref. CEI-37-829) and procedures were performed according to the European Union directives 63/2010UE and Spanish regulation RD 53/2013.

Male Sprague-Dawley rats (6 months old) were obtained from the Animal Quarters of the Universidad Autónoma de Madrid and housed in the Animal Facility of the Universidad Autónoma de Madrid (Registration number ES-20079-0000097), under 12 h light/dark cycles and standard feeding with fodder and water *ad libitum*. After 1 week of adaptation animals were fed a maintenance diet for rodents (Global Diet 2014, Harlan Laboratories Inc. Indianapolis, Indiana, USA) supplemented with fat (5%). The control group was supplemented with sunflower oil (5%) and the DHA group with 4.5% Marinol C-38 (Lipid Nutrition) and adjusted to 5% with sunflower oil. Nutrient content and energy distribution of each diet is summarized in Table 1. After 2 weeks on the diet, animals were divided into two groups: control and orchidectomized males. Male sex hormone deprivation was induced by orchidectomy at 18 weeks of age under anesthesia by isofluorane inhalation. Rats were treated with 0.30 mg/Kg meloxicam SC (Metacam 5 mg/ml; Boehringer-Ingelheim) immediately after surgery and with 50 mg/Kg ibuprofen, via oral administration for 4 days. Animals were maintained under experimental diets for six more weeks. At the end of the treatment, rats were sacrificed by CO_2_ inhalation and decapitation. The observation of seminal vesicles atrophy confirmed successful surgery. The mesenteric artery was carefully dissected out, cleaned of connective tissue and placed in Krebs-Henseleit solution (KHS) (containing, in mM: NaCl 115, CaCl_2_ 2.5, KCl 4.6, KH_2_PO_4_ 1.2, MgSO_4_ 1.2, NaHCO_3_ 25, glucose 11,1, Na_2_ EDTA 0.03) at 4°C.

**Table 1:** Nutrient and energy content of experimental diets.

	Control Diet	DHA Diet
Carbohydrates (g/100g)	59.84	59.84
Protein (g/100g)	14.39	14.39
Total Fat (g/100g)	9.19	9.19
DHA + EPA		2.01
Energy (kcal/100g)	271.78	271.78

### Blood pressure measurement

Systolic blood pressure was indirectly measured in awake animals by the tail-cuff method [17, 25] before and after the treatment using a Letica Digital Pressure Meter LE5000 (Barcelona, Spain).

### Release of prostanoids

After a stabilization period in KHS at 37°C for 30 minutes (pH 7.4), mesenteric rings from each group of rats was followed by 2 wash periods of 10 min using 0.2 mL of KHS. Once fresh KHS was replaced, after a period of 10 min and the medium was collected and stored at -80°C until used. Production of TXA_2_, PGI_2_ and PGE_2_, were monitored by measuring their stable metabolite TXB_2_, 6-keto-PGF_1α_, and PGE_2_, respectively, using the respective enzyme immunoassay kit (Cayman Chemical). Results were expressed as pg prostanoid/mL per mg of tissue.

### Production of nitric oxide

The fluorescent probe 4,5-diaminofluorescein was used to specifically evaluate NO production. Briefly, mesenteric segments from control, orchidectomized and orchidectomized with DHA diet groups were cryoprotected with 30% w/v sucrose in PBS, frozen and stored at -80°C. After a washing period with PBS, the artery segments were opened to uncover the artery lumen to allow a better penetrance of the probe. Then the segments were immersed in 4-(2-hydroxyethyl)-1-piperazineethanesulfonic acid (HEPES) buffer (in mM: NaCl 119, HEPES 20, CaCl_2_ 1.2, KCl 4.6, KH_2_PO_4_ 0.4, MgSO_4_ 1, NaHCO_3_ 5, glucose 5.5, Na_2_H_2_PO_4_ 0.15; pH 7.4) containing 4,5-diaminofluorescein (0.5 μM), and incubated in a light-protected, humidified chamber at 37°C for 45 min. Then, the segments were mounted on glass slides and imaged on a confocal microscope. Images were obtained with a LEICA (TCS ST2 DM IRE2) laser scanning confocal microscope (excitation 495 nm, emission 515 nm). Laser and image settings were unchanged for the acquisition of images from the three groups of rats. The photomicrographs show the intensity and location of 4,5-diaminofluorescein, which reflects NO production, so that comparison of these groups could be made. To analyze fluorescence intensity, the ImageJ Analysis Software (National Institutes of Health) was used. The amount of NO released was expressed as arbitrary units.

### Detection of superoxide anion

The fluorescent probe, hydroethidine, was used to evaluate superoxide anion levels *in situ*, as previously described [13, 26]. Mesenteric segments from the three groups were cryoprotected with 30% (w/v) sucrose in PBS, frozen and stored at -80°C. After a washing period with PBS, the artery segments were opened to uncover the artery lumen to allow for a better penetration of the probe. Then, the segments were immersed in HEPES buffer containing hydroethidine (5 μM), and incubated in a light-protected, humidified chamber at 37°C for 30 min. Segments were mounted on glass slides and imaged on a confocal microscope. Images were obtained with a LEICA (TCS ST2 DM IRE2) laser scanning confocal microscope (excitation 488 nm, emission 610 nm). Laser and image settings were unchanged for the acquisition of images from the three groups of rats. The photomicrographs show the intensity and location of hydroethidine, which reflects superoxide production, so that comparison of these groups could be made. To analyze fluorescence intensity, the ImageJ Analysis Software (National Institutes of Health) was used. The amount of superoxide formation was expressed as arbitrary units.

### Vascular reactivity

The method used for isometric tension recording has been previously described [26, 27]. In summary, mesenteric artery segments were suspended in an organ bath containing 5 mL of KHS at 37°C, continuously bubbled with 95% O_2_ -5% CO_2_ mixtures (pH 7.4). Two parallel stainless steel pins were introduced through the lumen of the vascular segment: one fixed to the bath wall and the other connected to a force transducer (Grass FTO3C; Grass Instruments Co., Quincy, MA, USA); this in turn was connected to a model 7D Grass polygraph. The activity was reflected in a computer through a computer program (eDAQ Software). The segments were subjected to a tension of 0.5 g which was re-adjusted every 15 min during a 90 min equilibration period before drug administration. After this, the vessels were exposed to KCl (75 mM) to check their functional integrity. After a washout period the viability of vascular endothelium was tested by the ability of 10 μM ACh to relax pre-contracted segments with 0.1 μM NA.

To determine the participation of innervation in the regulation of vascular tone in mesenteric artery, frequency-response curves to EFS were performed. The parameters used for EFS were 200 mA, 0.3 ms, 1–16 Hz, for 30 s with an interval of 1 min between each stimulus, the time required to recover basal tone. Since NO neurotransmitter and NA play key roles in the mesenteric artery, concentration-response curves to NA (1 nM-10 μM) and the NO donor, SNP (1 nM-10 μM) were performed.

Concentration-response curves to ACh (0.1 nM-10 μM) were performed in NA (0.1 μM) pre-contracted mesenteric artery rings from of the three groups of rats. To analyze the participation of NO and prostanoids on the ACh-induced response, the NO synthase inhibitor L-NAME (0.1 mM) or the nonselective inhibitor of COX-1/2, indomethacin (Indo, 10 μM), were added to the bath 30 minutes before performing the curve.

### Drugs

Drugs used were: L-NA hydrochloride, ACh chloride, L-NAME hydrochloride, indomethacin, potassium chloride, SNP (Sigma-Aldrich). Stock solutions (10 mM) of drugs were prepared in distilled water, except for NA which was dissolved in NaCl (0.9%)-ascorbic acid (0.01% w/v) solution, indomethacin in 1.5 mM NaHCO_3_ in dimethylsulfoxide. These solutions were maintained at -20°C and appropriate dilutions were made in KHS on the day of the experiment.

### Statistical analysis

Results are given as mean ± SEM (Standard Error of the Mean). The relaxation induced by ACh was expressed as a percentage of initial contraction elicited by NA. Statistical analysis was performed by comparing the curve obtained in the presence of the different substances with the control curve by means of two-way analysis of variance (ANOVA). For blood pressure, body weight, prostanoids, NO, and superoxide production, statistical analysis was done using Student’s *t*-test for unpaired experiments. A *p* value of less than 0.05 was considered significant.

## Results

### Animal weight and systolic blood pressure

Pre-diet body weight and blood pressure measures from control, orchidectomized and orchidectomized with the DHA-supplemented diet were not significantly modified. Six weeks post-diet all groups increased body weight to a similar extent and blood pressure did not show significant changes. Results already published [24].

### Production of nitric oxide

**Fig 1:**
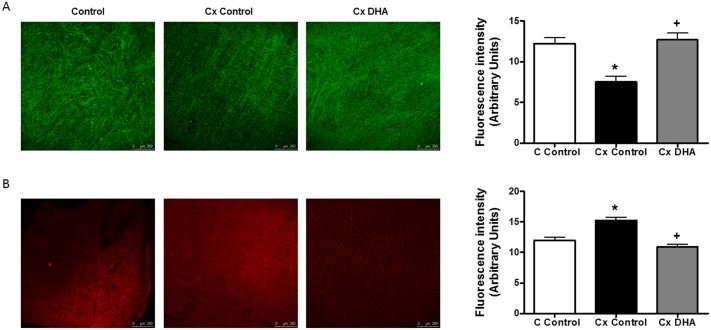
Effect of orchidectomy and DHA supplemented diet in endothelial production of NO and superoxide in rat mesenteric arteries. Confocal micrographs showing *in situ* detection of NO (A) or superoxide anion (B) in mesenteric artery segments from control (C) and orchidectomized (Cx) rats fed with a control diet and from Cx rats fed with a DHA-supplemented diet (Cx DHA). The sections shown are typical preparations from five rats. Quantitative analysis of fluorescence is also shown. Values are means ± SEMs, n = 5, *P < 0.001 compared with control animals; *+P* < 0.002 compared with Cx rats control diet.

The orchidectomized group showed a decrease in the fluorescence emitted by 4,5-diaminofluorescein after incubation in the mesenteric tissue with respect to arteries from control rats. DHA-supplemented diet restored the fluorescence levels reduced by orchiectomy similarly to values found in control animals (Fig 1A).

### Detection of superoxide anion

HE fluorescence levels showed an increase in orchidectomized rat vessels, compared to the ones from control group. The DHA-supplemented diet decreased these levels on the orchiectomized rats (Fig 1B).

### Release of prostanoids

**Fig 2:**
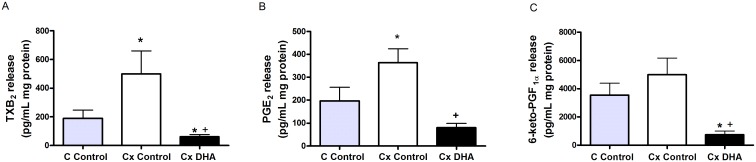
Effect of orchidectomy and DHA supplemented diet on prostanoid basal release in rat mesenteric arteries. Release of thromboxane B_2_ (TXB_2_), prostaglandin E_2_ (PGE_2_) and PGI_2_ (panels A-C) in mesenteric artery from control (C) and orchidectomized (Cx) rats fed with a control diet and from Cx rats fed with a DHA-supplemented diet (Cx DHA). Values are means ± SEMs. n = 4–8; *p <0.05 compared to arteries from control group, +*p <0*.*05* compared with arteries from Cx Control group.

Orchidectomy increased the basal release of TXB_2_ and PGE_2_ (Fig 2A and 2B). However, orchidectomy did not statistically modify the release of 6-keto-PGF_1α_ (Fig 2C). DHA supplemented diet decreased the release of the three prostanoids analyzed (Fig 2).

### Vascular reactivity

Mesenteric segments from all three groups contracted similarly to 75 mM KCl (C Control 1069 ± 78 mg; Cx Control 1291 ± 124 mg; Cx DHA 1199 ± 79 mg; *p* > 0.05).

**Fig 3:**
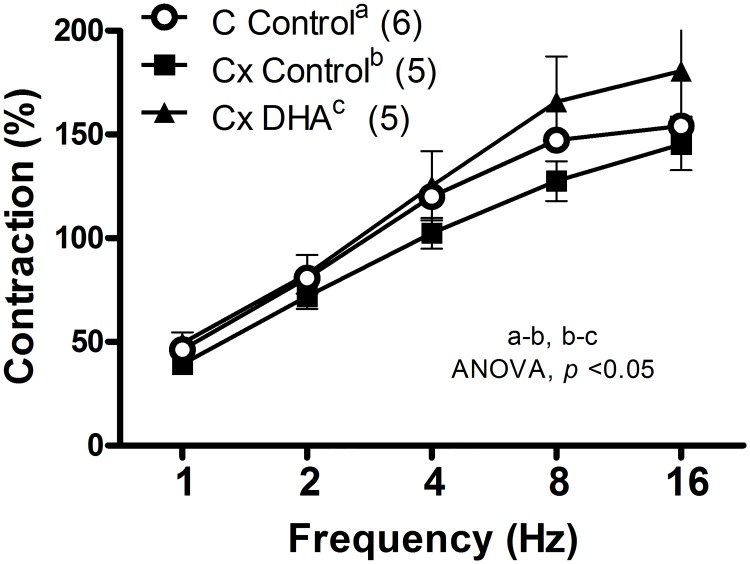
Effect of orchidectomy and DHA supplemented diet in the EFS-induced response in rat mesenteric arteries. Effect of EFS contractile response in mesenteric artery segments of male rats from control rats with control diet (A), orchidectomized rats with control diet (B) and orchidectomized rats with DHA-supplemented diet (C). Results (mean ± SEM) are expressed as percentage of contraction elicited by 75 mM KCl. Number of animals indicated in parenthesis.

**Fig 4:**
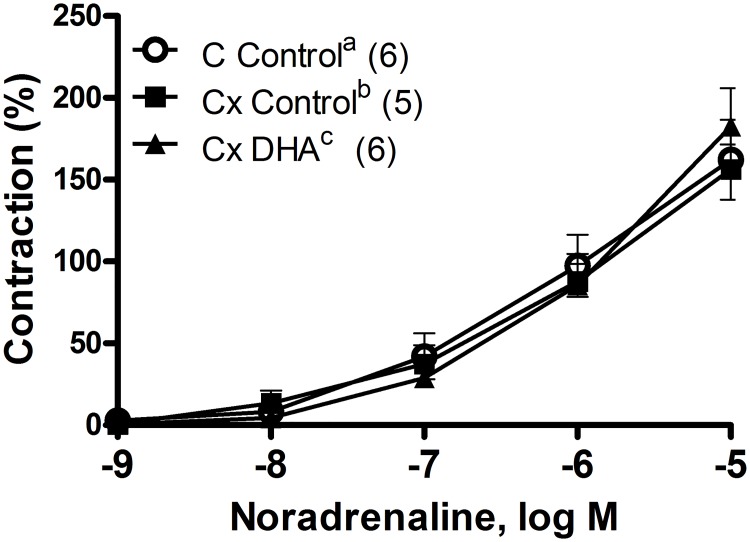
Effect of orchidectomy and DHA supplemented diet in the contractile response to NA curves in rat mesenteric arteries. Noradrenaline contractile response in mesenteric artery segments from control rats with control diet (A), orchidectomized rats with control diet (B) and orchidectomized rats with DHA-supplemented diet (C). Results (mean ± SEM) expressed as percentage of contraction elicited by 75 mM KCl. Number of animals indicated in parenthesis.

Orchidectomy decreased the EFS-induced contraction which was reversed by the DHA supplemented diet (Fig 3). The contractile response induced by exogenous NA (10 nM-10 μM) was similar in vessels from all three groups of rats (Fig 4).

**Fig 5:**
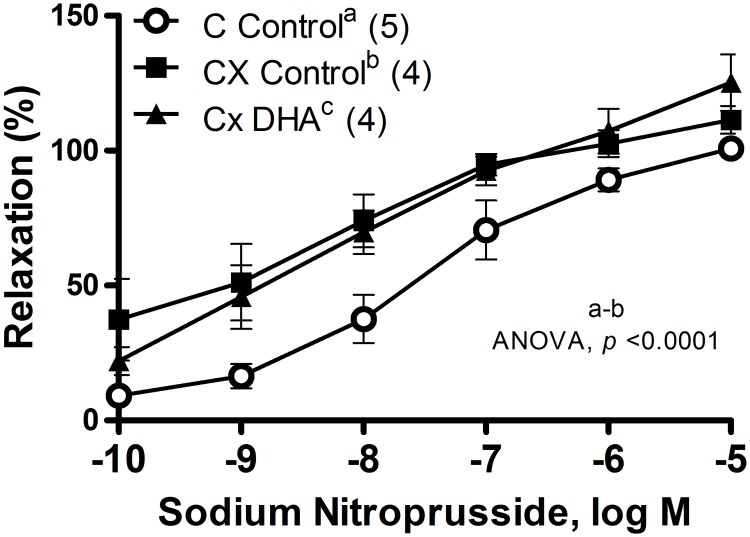
Effect of orchidectomy and DHA supplemented diet in the vasodilator response to SNP curves in rat mesenteric arteries. Orchidectomy and DHA supplemented diet influence on the vasodilator response to sodium nitroprusside in mesenteric artery segments of male rats, in control rats with control diet (A), orchidectomized rats with control diet (B) and orchidectomized rats with DHA-supplemented diet (C). Results (mean ± SEM) are expressed as percentage of inhibition of precontraction elicited by 1 μM noradrenaline. Number of animals indicated in parenthesis.

**Fig 6:**
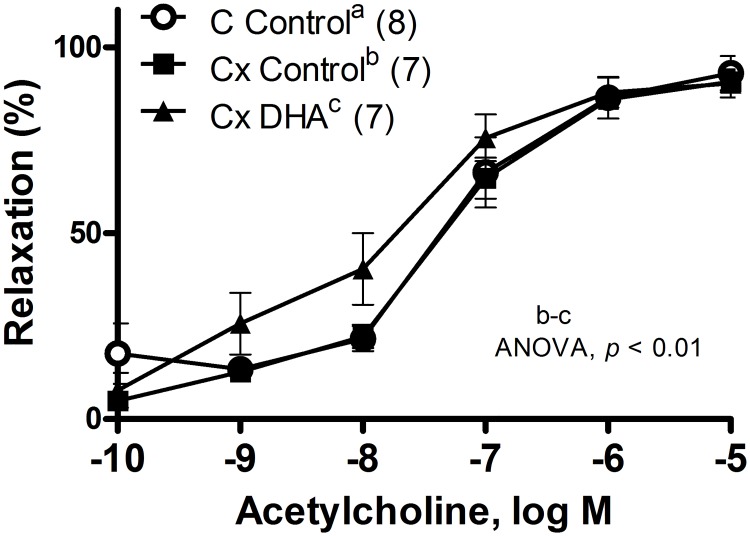
Effect of orchidectomy and DHA supplemented diet in the vasodilator response to ACh curves in rat mesenteric arteries. Concentration-response curves to acetylcholine in mesenteric artery segments from control (C) and orchidectomized (Cx) rats fed with a control diet and from Cx rats fed with a DHA-supplemented diet (Cx DHA). Results (means + SEMs) are expressed as percentage of inhibition of the contraction induced by 1 μM noradrenaline. The number of animals is indicated in parenthesis.

The relaxation induced by SNP was analyzed in segments previously pre-contracted with NA (1 μM). Orchidectomy significantly increased the vasodilator response in segments from orchidectomized rats (ANOVA, *p* < 0.001, Fig 5). This response was not modified by DHA supplemented diet (Fig 5). The vasodilator response induced by ACh (0.1 nM—10 μM) in segments pre-contracted with NA (1 μM) was not modified by orchidectomy, while the DHA supplemented diet increased this response (ANOVA, *p* < 0.01, Fig 6).

**Fig 7:**
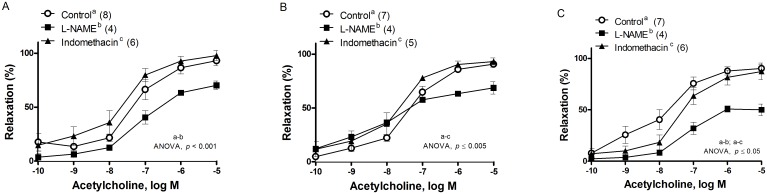
Effect of L-NAME and indomethacin in the ACh-induced vasodilator response in rat mesenteric arteries. Effect of L-NAME (0.1 mM) or indomethacin (Indo, 10 μM) on the concentration response curves to acetylcholine in the NA-pre-contracted mesenteric artery segments from control rats with control diet (A), orchidectomized rats with control diet (B) and orchidectomized rats with DHA-supplemented diet (C). Results (means ± SEMs) are expressed as percentage of inhibition of the contraction induced by 1 μM noradrenaline. The number of animals is indicated in parenthesis.

The effect of castration and DHA supplemented diet in this stage on NO contribution was studied in presence of the NOS inhibitor, L-NAME, which decreased the ACh induced response in arteries from the control group (ANOVA, *p* < 0.001, Fig 7A), did not modify those of orchidectomized rats (ANOVA, *p* > 0.05, Fig 7B), whereas it decreased Cx DHA group (ANOVA, *p*<0.0001, Fig 7C). Incubation with the COX inhibitor, indomethacin (10 μM), tended to increase the ACh-induced response in control arterial segments (ANOVA, *p* = 0.057 7-A), while increased the vasorelaxation in the orchidectomized group (ANOVA, *p* = 0.004, Fig 7B), and decreased it in Cx DHA group (ANOVA, *p* = 0.02, Fig 7C).

## Discussion

There is compelling evidence which demonstrates the beneficial effect of n-3 PUFAs in vascular dysfunction for the prevention and treatment of cardiovascular disorders [21, 22]. Although the relationship between decreased levels of sex hormones and increased incidence of cardiovascular disease is established [28, 29], to date there are no studies examining the effect of diet supplemented with DHA when gonadal function is lost. Recently, we have reported positive modifications induced by a DHA-supplemented diet on lipid profile, redox status and vasodilator function of aorta from orchidectomized rats [24]. Taking into account that the mesenteric vascular bed importantly contributes to the control of blood pressure, in the present study we have focused on analyzing the effect of DHA-diet in the vascular function of rat mesenteric artery from orchidectomized rats.

We previously published that the orchidectomy maintained for 5 months induces an increase on prostanoid release in mesenteric artery [30, 31] and aorta [17, 25], due to an oxidative stress augmentation. The results now presented are in agreement with these previous findings, since TXA_2_, PGI_2_, and PGE_2_ are increased from the 6 week post-orchidectomy, as observed in aorta [24] and in mesenteric artery for the case of TXA_2_ [32]. However, DHA-supplemented diet decreased prostanoids release near to the control group levels. According with this data, the consumption of omega-3 fatty acid has been reported to inhibit the nuclear factor kappa B activation and of COX-2 expression [33], and to decrease the production of prostanoids [34]. Previous publications demonstrate that DHA competes with other PUFAs, such as the arachidonic acid, resulting in a decrease of prostanoid synthesis [35] and in an anti-inflammatory effect.

NO release was decreased by castration, as reported earlier in aortas from 6 weeks post-orchidectomized rats [32, 24]. The influence of the DHA-supplemented diet, showed a restoration in NO release levels, closely to the ones from the control group, as previously found in aorta [24]. However, DHA has been reported not to modify the release of NO in aged [36] and hypertensive [37] rats, indicating that the choice of the experimental model is crucial.

NO bioavailability is strongly determined by the level of oxidative stress. It is already known that the loss of gonadal function increases the production of ROS [13, 20], like superoxide anion, a source of many other reactive oxygen intermediates, which quench NO. The results obtained in the present study show increased superoxide anion production in mesenteric artery from orchidectomized rats, which is in agreement with previous publications [13, 19], and that demonstrate the beneficial effects from male sex hormones in vascular function. Supplementation with the DHA diet diminished the superoxide anion levels near to control levels, similar to those reported in the aorta of these animals [24]. In line with these results, is the ability of n-3 PUFAs to decrease superoxide anion production in mouse aorta [38] and human fibroblasts [39]. In addition, DHA has been reported to decrease oxidative stress in vascular [40], nervous [41] and immune [42] systems. All these data demonstrate the antioxidant properties of DHA in situations where oxidative stress is increased.

Taking into account that: i) NO, prostanoids and ROS are able to modulate the vascular tone, ii) the DHA-supplement diet restores the altered production of these factors in orchidectomized rats, and iii) the important contribution of mesenteric artery in the control of systemic vascular resistance, the vasoconstrictor response mediated by the neurotransmitters released after EFS and the endothelium-dependent vasodilator response were analyzed.

We observed that orchidectomy decreased the EFS-induced contraction, which is in agreement with that observed in mesenteric artery from 5 months-post-orchidectomized rats [13] in which the release of NA was not modified [18]. DHA-supplemented diet reversed the reduction in EFS-induced contractile response caused by orchidectomy. These actions seem to be specific on EFS-induced contraction since the contraction induced by 75 mM of KCl was not modified by the orchidectomy or DHA-diet. In this regard, the contractile response induced by potassium depolarization was not altered by a DHA diet in hypertensive rats [37]. Although, we have previously reported that the orchidectomy did not modify the release of neuronal NO [13] and NA [18], it would have been desirable to determine the neurogenic release of NA and NO. The authors acknowledge this limitation of the current study and, because of the intriguing and complex interactions among the different factors, this issue will be addressed in future studies. Nevertheless, it has been reported that DHA contained in fish oil did not significantly alter neuronal and cardiovascular control in normotensive and prehypertensive humans [43], while eicosapentanoic acid supplementation reduced cardiac noradrenaline concentration in diabetic rats [44], indicating that the cardiovascular benefits of omega-3 depend on the initial stage of the pathology. Since 5 months post-orchidectomy modified the vasomotor responses induced by NO and NA, these responses were analyzed in mesenteric arteries after 6 weeks post-orchidectomy fed control and DHA-supplemented diet. Orchidectomy did not produce any relevant influence on NA-induced contractile response, suggesting that this castration period does not interfere with the response once released the neurotransmitter, which is consistent with some publications [45]. However, it contrasts with other studies in which orchidectomy decreased [13, 46] or increased [47] NA-induced contractile response. These variations might be attributed to castration and/or the animal model employed as mentioned above. Additionally, the DHA-supplemented diet did not influence NA sensibility in smooth muscle cells, since its response was similar to that produced by orchidectomy. This result is in agreement with studies describing no modification by DHA on the contractile response after alpha-adrenoceptors activation [48, 49]. Regarding the vasodilator effect of NO, the SNP-induced response was analyzed. The vasodilator response was significantly increased by orchidectomy, which agrees with reports on mesenteric arteries of rats after 5 months post-orchidectomy [31]. This response was not modified by the DHA supplemented diet, meaning that PUFAs have no effect on NO sensitivity in smooth muscle cell, as it was found in aortic segments [24] as published earlier [36]. Therefore, one possible explanation for the recovering of the decreased EFS-induced response in orchidectomized rats fed with DHA, could be the decline in the oxidative stress and, in turn, the decreased vasodilator effect from the NO-derived products [13]. However, the participation of different mediators cannot be discarded, which would be the goal of future research.

As stated before, the endothelium-dependent relaxation was also studied. In this regard, the ACh-induced response was unmodified by orchidectomy, as previously found in the aorta of these animals [24]. On the other hand, the diet supplemented with DHA significantly increased the endothelium dependent relaxation, caused by reduced oxidative stress, which increases NO bioavailability, as demonstrated by the levels of O_2_^-^ and NO found in these arteries, and in aorta from a previous study [24].

To associate the participation of endothelial NO in the ACh- induced response, it was analyzed by using L-NAME, a NOS inhibitor. The decreased vasodilator response induced by ACh in the presence of L-NAME was grater in the rats fed with the DHA supplemented diet than in control and orquidectomized groups, evidencing that DHA increases NO participation. This response can be related to an increase of eNOS activation [50] aside from the increase in NO bioavailability, through the reduction of superoxide anion formation. The lack of change in the response from the orchidectomized group indicates a reduction in the participation of NO and the involvement from other vasodilator mechanisms apart from NO, such as prostanoids, when this is inhibited. Therefore, the role of prostanoids in ACh response was elucidated by incubation with the COX inhibitor, indomethacin. The increased response in orchidectomy suggests the involvement of vasoconstrictor prostanoids. For this reason, when prostanoids are removed by indomethacin the relaxation to ACh is greater, although the participation of other vasodilator factors cannot be discarded. However, in the DHA group vasodilator prostanoids are predominant, so ACh induced relaxation is smaller in the presence of indomethacin which suggests that the diet does improve more vasodilator than vasoconstrictor prostanoids. This is contrary to results found in aorta, where there was no modification of this response [24], indicating that the vascular effect of DHA in the relaxing response mediated by prostanoids depends on the vascular bed. It has been reported that when prostanoid synthesis is inhibited, NO synthesis is modified [25] and viceversa; also, other factors could be working to compensate the loss of prostanoids [18].

In summary, orchidectomy was associated with endothelial dysfunction of mesenteric artery caused from increased oxidative stress, by means of an augmented formation of prostanoids and superoxide production, and a reduction in endothelial NO formation. Also, the neurogenic response was decreased. The DHA-supplemented diet decreased prostanoid and superoxide levels, at the same time that increased NO formation and bioavailability in orchidectomized rats, and recovered the neurogenic response that may account for a better regulation of vascular function. The results obtained in this study together with others previously reported [24] suggest that a DHA-supplemented diet may be helpful to treat cardiovascular disease, since this PUFA exerts beneficial effects, in addition to the lipid profile, on the function of both conduit and resistance arteries.

## Supporting Information

S1 File Individual experimental data points for Fig 1A and 1B. (XLSX)

S2 File Individual experimental data points for Fig 2A, 2B and 2C. (XLSX)

S3 File Individual experimental data points for Fig 3. (XLSX)

S4 File Individual experimental data points for Fig 4. (XLSX)

S5 File Individual experimental data points for Fig 5. (XLSX)

S6 File Individual experimental data points for Fig 6. (XLSX)

S7 File Individual experimental data points for Fig 7A, 7B and 7C. (XLSX)

## References

[1] Félétou M, Vanhoutte PM. Endothelium-dependent hyperpolarization of canine coronary smooth muscle. *Br J Pharmacol*. 1988; 93: 515–524. 2453240 10.1111/j.1476-5381.1988.tb10306.x PMC1853853

[2] Orshal JM, Khalil RA. Gender, sex hormones and vascular tone. *Am J Physiol Regul Integr Comp Physiol*. 2004; 286: R233–R249. 10.1152/ajpregu.00338.2003 14707008

[3] Lincoln TM, Dey N, Sellak H. CGMP-dependent protein kinase signaling mechanisms in smooth muscle: From the regulation of tone to gene expression. *J Appl Physiol*. 2001; 91: 1421–1430. 11509544 10.1152/jappl.2001.91.3.1421

[4] Munzel T, Heitzer T, Harrison DG. The physiology and pathophysiology of the nitric oxide/superoxide system. *Herz*. 1997; 22: 158–172. 9232165 10.1007/BF03044353

[5] Kukreja RC, Kontos HA, Hess ML, Ellis EF. PGH synthase and lipoxygenase generate superoxide in the presence of NADH or NADPH. *Circ Res*. 1986; 59: 612–619. 3028671 10.1161/01.res.59.6.612

[6] Mueller CFH, Laude K, McNally JS, Harrison DG. Redox mechanisms in blood vessels. *Arterioscler Thromb Vasc Biol*. 2005; 25: 274–278. 10.1161/01.ATV.0000149143.04821.eb 15514203

[7] Cai H, Harrison DG. Oxidant Stress in Cardiovascular Diseases. *Circ Res*. 2000; 87: 840–844. 11073878 10.1161/01.res.87.10.840

[8] Vane JR, Bakhle YS, Botting RM. Cyclooxygenase 1 and 2. Annu Rev Pharmacol Toxicol. 1998; 38: 97–120. 10.1146/annurev.pharmtox.38.1.97 9597150

[9] Sellers MM, Stallone JN. Sympathy for the devil: the role of thromboxane in the regulation of vascular tone and blood pressure. Am J Physiol. 2008; 294: H1978–H1986. 10.1152/ajpheart.01318.2007 18310512

[10] Li YJ, Duckles SP. Effect of endothelium on the actions of sympatheticic and sensory nerves in the perfused rat mesentery. Eur J Pharmacol. 1992; 210: 23–30. 1376271 10.1016/0014-2999(92)90647-m

[11] Boeckstaens GE, Pelckmans PA. Nitric Oxide and the non-adrenergic non-choligernic neurotransmission. Comp Biochem Physiol. 1997; 118: 925–937. 10.1016/s0300-9629(97)00022-4 9505411

[12] Marín J, Balfagón G. Effect of clenbuterol on non endotelial nitric oxide release in rat mesenteric arteries and the involvement of beta-adrenoreceptors. Br J Pharmacol. 1998; 123: 473–478. 9647470 10.1038/sj.bjp.0701856 PMC1565411

[13] Martín MC, Balfagón G, Minoves N, Blanco-Rivero J, Ferrer M. Androgen deprivation increases neuronal nitric oxide metabolism and its vasodilator effect in rat mesenteric arteries. Nitric Oxide 2005; 12:163–176. 15875321 10.1016/j.niox.2005.02.003

[14] Marín J, Ferrer M, Balfagón G. Role of protein kinase C in electrical-stimulation-induced neuronal nitric oxide release in mesenteric arteries from hypertensive rats. Clin Sci (Lond). 2000; 99: 277–283. 10995592

[15] del Campo L, Ferrer M, Balfagón G. Hypertension alters the function of nitrergic and sensory innervation in mesenteric arteries from female rats. J Hypertens. 2009; 27: 791–799. 10.1097/HJH.0b013e32832531e6 19516178

[16] Jones RD, Hugh Jones T, Channer KS. The influence of testosterone upon vascular reactivity. Eur J Endocrinol. 2004; 151: 29–37. 15248819 10.1530/eje.0.1510029

[17] Martorell A, Blanco-Rivero J, Aras-López R, Sagredo A, Balfagón G, Ferrer M. Orchidectomy increases the formation of prostanoids and modulates their role in the acetylcholine-induced relaxation in the rat aorta. Cardiovasc Res. 2008; 77: 590–599. 10.1093/cvr/cvm059 18006440

[18] del Campo L, Sagredo A, Aras-López R, Balfagón G, Ferrer M. Orchidectomy increases the formation of non-endothelial thromboxane A(2) and modulates its role in the electrical field stimulation-induced response in rat mesenteric artery. J Endocrinol. 2008; 197: 371–379. 10.1677/JOE-07-0647 18434367

[19] Blanco-Rivero J, Sagredo A, Balfagón G, Ferrer M. Orchidectomy increases expression and activity of Cu/Zn-superoxide dismutase, while decreasing endothelial nitric oxide bioavailability. J Endocrinol. 2006; 190: 771–8. 10.1677/joe.1.06887 17003278

[20] Ferrer M, Tajera N, Marín J, Balfagón G. Androgen deprivation facilitates acetilcholyne-induced relaxation by superoxide anion generation. Clin Sci. 1999; 97: 625–631. 10585889

[21] Mori TA, Watts GF, Burke V, Hilme E, Puddey IB, Beilin LJ. Differential effects of eicosapentaenoic acid and docosahexaenoic acid on vascular reactivity of the forearm microcirculation in hyperlipidemic, overweight men. Circulation 2000; 102: 1264–1269. 10982541 10.1161/01.cir.102.11.1264

[22] Mozaffarian D, Geelen A, Brouwer IA, Geleijnse JM, Zock PL, Katan MB. Effect of fish oil on heart rate in humans: a meta-analysis of randomized controlled trials. Circulation 2005;112: 1945–1952. 10.1161/CIRCULATIONAHA.105.556886 16172267

[23] Gortan Cappellari G, Losurdo P, Mazzucco S, Panizon E, Jevnicar M, Macaluso L, et al Treatment with n-3 polyunsaturated fatty acids reverses endothelial dysfunction and oxidative stress in experimental menopause. J Nutr Biochem. 2013; 24: 371–379. 10.1016/j.jnutbio.2012.07.012 23159066

[24] Villalpando DM, Navarro R, Del Campo L, Largo C, Muñoz D, Tabernero M, et al Effect of Dietary Docosahexaenoic Acid Supplementation on the Participation of Vasodilator Factors in Aorta from Orchidectomized Rats. PLoS One. 2015;10(11):e0142039 10.1371/journal.pone.0142039 26540339 PMC4634962

[25] Martorell A, Sagredo A, Aras-López R, Balfagón G, Ferrer M. Ovariectomy increases the formation of prostanoids and modulates their role in acetylcholine-induced relaxation and nitric oxide release in the rat aorta. Cardiovasc Res. 2009; 84: 300–308. 10.1093/cvr/cvp214 19567483

[26] Sagredo A, del Campo L, Martorell A, Navarro R, Martín MC, Blanco-Rivero J, Ferrer M. Ovariectomy increases the participation of hyperpolarizing mechanisms in the relaxation of rat aorta. PLoS One 2013;8:e73474 10.1371/journal.pone.0073474 24058477 PMC3772950

[27] Nielsen KC, Owman C. Contractile response and amine receptor mechanism in isolated middle cerebral artery of the cat. Brain Res. 1971; 27:33–42. 4396591 10.1016/0006-8993(71)90370-2

[28] Barrett-Connor E, Khaw KT. Endogenous sex hormones and cardiovascular disease in men. A prospective population-based study. Circulation 1988; 78: 539–545. 3409497 10.1161/01.cir.78.3.539

[29] Kelly DM, Jones TH. Testosterone: a vascular hormone in health and disease. J Endocrinol. 2013; 217: R47–R71. 10.1530/JOE-12-0582 23549841

[30] Blanco-Rivero J, Balfagón G, Ferrer M. Orchidectomy modulates alpha2-adrenoceptor reactivity in rat mesenteric artery through increased thromboxane A2 formation. J Vasc Res. 2006; 43: 101–118. 10.1159/000089791 16293968

[31] Blanco Rivero J, Sagredo A, Balfagón G, Ferrer M. Protein kinase C activation increases endothelial nitric oxide release in mesenteric arteries from orchidectomized rats. J Endocrinol. 2007; 192: 189–197. 10.1677/joe.1.07079 17210756

[32] del Campo M, Sagredo A, Del Campo L, Villalobo A, Ferrer M. Time-dependent effect of orchidectomy on vascular nitric oxide and thromboxane A2 release. Functional implications to control cell proliferation through activation of the epidermal growth factor receptor. PLoS One. 2014; 9(7):e102523 10.1371/journal.pone.0102523 25013941 PMC4094513

[33] Matsumoto T, Nakayama N, Ishida K, Kobayashi T, Kamata K. Eicosapentaenoic acid improves imbalance between vasodilator and vasoconstrictor actions of endothelium-derived factors in mesenteric arteries from rats at chronic stage of type 2 diabetes. J Pharmacol Exp Ther. 2009; 329: 324–34. 10.1124/jpet.108.148718 19164460

[34] Farré LA, Macaya C. Antithrombotic and antiinflammatory effects of omega-3 fatty acids. Rev Esp Cardiol Supl. 2006; 6: 31D–7D.

[35] Wada M, DeLong CJ, Hong YH, Rieke CJ, Song I, Sidhu RS, et al Enzymes and receptors of prostaglandin pathways with arachidonic acid-derived versus eicosapentaenoic acid-derived substrates and products. J Biol Chem. 2007; 282: 22254–22266. 10.1074/jbc.M703169200 17519235

[36] Hashimoto M, Shinozuka K, Gamoh S, Tanabe Y, Hossain MS, Kwon YM, et al The hypotensive effect of docosahexaenoic acid is associated with the enhanced release of ATP from the caudal artery of aged rats. J Nutr. 1999; 129: 70–76. 9915878 10.1093/jn/129.1.70

[37] Casos K, Zaragoza MC, Zarkovic N, Zarkovic K, Andrisic L, Portero-Otin M, et al A fish-oil-rich diet reduces vascular oxidative stress in apoE (−/−) mice. Free Radic Res. 2010; 44: 821–929. 10.3109/10715762.2010.485992 20528577

[38] Rossary A, Arab K, Steghens JP. Polyunsaturated fatty acids modulate NOX 4 anion superoxide production in human fibroblasts. Biochem J. 2007; 406: 77–83. 10.1042/BJ20061009 17472580 PMC1948982

[39] Burban M, Meyer G, Olland A, Séverac F, Yver B, Toti F, et al An Intravenous Bolus of EPA: DHA 6: 1 Protects Against Myocardial Ischemia-Reperfusion-Induced Shock. Shock 2016; 46: 549–556. 10.1097/SHK.0000000000000624 27058043

[40] Zarrouk A, Nury T, Samadi M, O'Callaghan Y, Hammami M, O'Brien NM, et al Effects of cholesterol oxides on cell death induction and calcium increase in human neuronal cells (SK-N-BE) and evaluation of the protective effects of docosahexaenoic acid (DHA; C22:6 n-3). Steroids 2015; 99: 238–247. 10.1016/j.steroids.2015.01.018 25656786

[41] Pettit LK, Varsanyi C, Tadros J, Vassiliou E. Modulating the inflammatory properties of activated microglia with Docosahexaenoic acid and Aspirin. Lipids Health Dis. 2013; 12: 16 10.1186/1476-511X-12-16 23398903 PMC3663775

[42] Engler MM, Engler MB, Pierson DM, Molteni LB, Molteni A. Effects of docosahexaenoic acid on vascular pathology and reactivity in hypertension. Exp Biol Med. (Maywood). 2003; 228: 299–307. 12626775 10.1177/153537020322800309

[43] Carter JR, Schwartz CE, Yang H, Joyner MJ. Fish oil and neurovascular control in humans. Am J Physiol. 2012; 303:H450–H456. 10.1152/ajpheart.00353.2012 PMC3423144 22707560

[44] Nishimura M, Nanbu A, Komori T, Ohtsuka K, Takahashi H, Yoshimura M. Eicosapentaenoic acid stimulates nitric oxide production and decreases cardiac noradrenaline in diabetic rats. Clin Exp Pharmacol Physiol. 2000; 27: 618–624. 10901392 10.1046/j.1440-1681.2000.03311.x

[45] Chen DC, Duckles SP, Krause DN. Postjunctional alpha2-adrenoceptors in the rat tail artery: effect of sex and castration. Eur J Pharmacol. 1999; 372: 247–252. 10395019 10.1016/s0014-2999(99)00226-5

[46] Cingarrella A, Bolego C, Pinna C, Zanardo R, Nardi F, Zancan V, Puglisi L. Androgen deprivation, estrogen treatment and vascular function in male rat aorta. Naunyn Scmiedebergs Arch Pharmacol. 2000; 361: 166–172. 10.1007/s002109900173 10685872

[47] Siddiqui A, Shah BH. Neonatal androgen manipulation differentially affects the development of monoamine systems in rat cerebral cortex, amygdala and hypothalamus. Brain Res Dev Brain Res. 1997; 98: 247–252. 9051266 10.1016/s0165-3806(96)00171-x

[48] Otsuka K, Tanaka Y, Tanaka H, Koike K, Shigenobu K. Comparison of the inhibitory effects of docosahexaenoic acid (DHA) on U46619- and phenylephrine-induced contractions in guinea-pig aorta. Biol Pharm Bull. 2005; 28: 1298–300. 15997118 10.1248/bpb.28.1298

[49] Sato K, Chino D, Kobayashi T, Obara K, Miyauchi S, Tanaka Y. Selective and potent inhibitory effect of docosahexaenoic acid (DHA) on U46619-induced contraction in rat aorta. J Smooth Muscle Res. 2013; 49: 63–77. 24304639 10.1540/jsmr.49.63 PMC5137318

[50] Omura M, Kobayashi S, Mizukami Y, Mogami K, Todoroki-Ikeda N, Miyake T, et al Eicosapentaenoic acid (EPA) induces Ca(2+)-independent activation and translocation of endothelial nitric oxide synthase and endothelium-dependent vasorelaxation. FEBS Lett. 2001; 487: 361–366. 11163359 10.1016/s0014-5793(00)02351-6

